# Evasion of a Human Cytomegalovirus Entry Inhibitor with Potent Cysteine Reactivity Is Concomitant with the Utilization of a Heparan Sulfate Proteoglycan-Independent Route of Entry

**DOI:** 10.1128/JVI.02012-19

**Published:** 2020-03-17

**Authors:** M. J. Murray, N. I. Bonilla-Medrano, Q. L. Lee, S. J. Oxenford, R. Angell, D. P. Depledge, M. B. Reeves

**Affiliations:** a Institute of Immunity and Transplantation, UCL, Royal Free Hospital Campus, London, United Kingdom; b Translational Research Office, School of Pharmacy, UCL, London, United Kingdom; c Department of Microbiology, New York University School of Medicine, New York, New York, USA; Northwestern University

**Keywords:** antibody function, antiviral agents, cytomegalovirus, virus entry

## Abstract

The dependence of viruses on the host cell to complete their replicative cycle renders cellular functions potential targets for novel antivirals. We screened a panel of broadly acting cellular ion channel inhibitors for activity against human cytomegalovirus (HCMV) and identified the voltage-gated chloride ion channel inhibitor 4,4′-diisothiocyano-2,2′-stilbenedisulfonic acid (DIDS) as a potent inhibitor of HCMV replication. Time-of-addition studies demonstrated that DIDS inhibited entry via direct interaction with the virion that impeded binding to the plasma membrane. Synthesis and analysis of pharmacological variants of DIDS suggested that intrinsic cysteine, and not lysine, reactivity was important for activity against HCMV. Although sequencing of DIDS-resistant HCMV revealed enrichment of a mutation within UL100 (encoding glycoprotein M) and a specific truncation of glycoprotein RL13, these did not explain the DIDS resistance phenotype. Specifically, only the introduction of the RL13 mutant partially phenocopied the DIDS resistance phenotype. Serendipitously, the entry of DIDS-resistant HCMV also became independent of heparan sulfate proteoglycans (HSPGs), suggesting that evasion of DIDS lowered dependence on an initial interaction with HSPGs. Intriguingly, the DIDS-resistant virus demonstrated increased sensitivity to antibody neutralization, which mapped, in part, to the presence of the gM mutation. Taken together the data characterize the antiviral activity of a novel HCMV inhibitor that drives HCMV infection to occur independently of HSPGs and the generation of increased sensitivity to humoral immunity. The data also demonstrate that compounds with cysteine reactivity have the potential to act as antiviral compounds against HCMV via direct engagement of virions.

**IMPORTANCE** Human cytomegalovirus (HCMV) is major pathogen of nonimmunocompetent individuals that remains in need of new therapeutic options. Here, we identify a potent antiviral compound (4,4′-diisothiocyano-2,2′-stilbenedisulfonic acid [DIDS]), its mechanism of action, and the chemical properties required for its activity. In doing so, the data argue that cysteine-reactive compounds could have the capacity to be developed for anti-HCMV activity. Importantly, the data show that entry of DIDS-resistant virus became independent of heparan sulfate proteoglycans (HSPGs) but, concomitantly, became more sensitive to neutralizing antibody responses. This serendipitous observation suggests that retention of an interaction with HSPGs during the entry process *in vivo* may be evolutionarily advantageous through better evasion of humoral responses directed against HCMV virions.

## Abstract (precis)

Human cytomegalovirus (HCMV) is major pathogen of nonimmunocompetent individuals that remains in need of new therapeutic options. Here, we identify a potent antiviral compound (4,4′-diisothiocyano-2,2′-stilbenedisulfonic acid [DIDS]), its mechanism of action, and the chemical properties required for its activity. In doing so, the data argue that cysteine-reactive compounds could have the capacity to be developed for anti-HCMV activity. Importantly, the data show that entry of DIDS-resistant virus became independent of heparan sulfate proteoglycans (HSPGs) but, concomitantly, became more sensitive to neutralizing antibody responses. This serendipitous observation suggests that retention of an interaction with HSPGs during the entry process *in vivo* may be evolutionarily advantageous through better evasion of humoral responses directed against HCMV virions.

## INTRODUCTION

Human cytomegalovirus (HCMV) entry involves a complex series of events requiring the interaction of virus-encoded glycoproteins with cell membrane receptors (1–10). The ability of HCMV to utilize multiple entry pathways likely underpins its ability to infect a number of different cell types. As such, HCMV entry has been reported to proceed via pH-dependent fusion, endocytosis, and macropinocytosis in a cell-type- and virus strain-specific manner (11–14). Prior to the cell-type-specific events that dictate HCMV tropism, the virus engages with the cell via a low-affinity interaction with heparan sulfate proteoglycans (HSPGs) (1, 2, 15). The importance of this interaction for HCMV entry is emphasized by the ability of heparin to act as a potent inhibitor of HCMV infection (2). Indeed, many viruses utilize an interaction with HSPGs, presumably as a mechanism to establish initial contact with the plasma membrane prior to triggering the complicated entry process (16–18).

The requirement for virus-encoded glycoproteins to drive entry exposes the virus to humoral immunity. Antibodies that recognize glycoprotein B (gB), gH, gL, and gO and the pentameric complex have all been shown to be potent inhibitors of viral infection *in vitro* (19–26). Furthermore, promising data from clinical studies demonstrate that passive immunization with antibodies directed against viral complexes incorporating gH/gL can reduce HCMV viremia *in vivo* (27). Antibody-mediated neutralization of HCMV can occur either via the recruitment of complement to promote pathogen lysis or through steric hindrance of the interactions of these glycoproteins with their cell surface receptors (28). Although the specific interactions that occur at the cell surface are not fully understood, a substantial body of work points toward cell-type-specific roles for the gH/gL complexes, whereas gB is involved in entry into all cell types with reported roles in initial attachment, fusion and receptor binding, and activation of signaling pathways (4, 29–34).

The other angle of viral entry is the role of host cell functions in the process. Recently, a number of studies have pointed to the importance of ion channel activity in the entry of a range of viruses, with roles in entry and uncoating being reported for diverse viruses, such as influenza A virus, Ebola virus, and Bunyamwera virus (35–38). Furthermore, the impact of ion channels is not restricted to the viral entry process, with roles for potassium channels K_v_2.1 and TASK-1 demonstrated in the activation of apoptosis induced by hepatitis C virus (HCV) and the budding of HIV-1, respectively (39, 40). The fact that a number of ion channel inhibitors are used to treat nonviral disorders in the clinic has raised the possibility that a number of licensed compounds with good safety profiles might be repurposed for use in viral infections (41).

Here, we report the unexpected outcome of a compound screen of known ion channel inhibitors. Further characterization of a lead hit (4,4′-diisothiocyano-2,2′-stilbenedisulfonic acid [DIDS]) revealed that the inhibitory activity was not related to inhibition of its canonical ion channel target but instead, upon characterization, revealed that the compound directly bound the virus to inhibit initial viral engagement at the stage of HSPG interactions. Further characterization of the mechanism of action revealed that DIDS inhibition was dependent on a reversible cysteine-dependent interaction with the virion to prevent entry. Generation of a DIDS-resistant mutant revealed that DIDS resistance was concomitant with resistance to heparin and an entry pathway independent of HSPGs. Surprisingly, the acquisition of a DIDS/heparin-resistant phenotype resulted in increased sensitivity to neutralizing antibody responses present in sera when tested *in vitro*. Sequence analysis of the mutant revealed enrichment for mutations in two genes—RL13 and UL100—that encode glycoproteins present in the virion envelope (42, 43). Interestingly, reengineering of these mutations into the strain Merlin bacterial artificial chromosome (BAC) revealed a complicated phenotype. Partial resistance to DIDS was imparted by the RL13 truncation, but intriguingly, it was reduced in the presence of the gM mutation. However, it was the gM and not the RL13 mutation that appeared to be the major contributor to the antibody sensitivity phenotype of the DIDS-resistant virus. In summary, the data also suggest that resistance to the action of the antiviral entry inhibitor DIDS may be countered by increased sensitivity to humoral immunity, which could have positive implications for the potential development and use of DIDS-like molecules against HCMV *in vivo*.

## RESULTS

**FIG 1:**
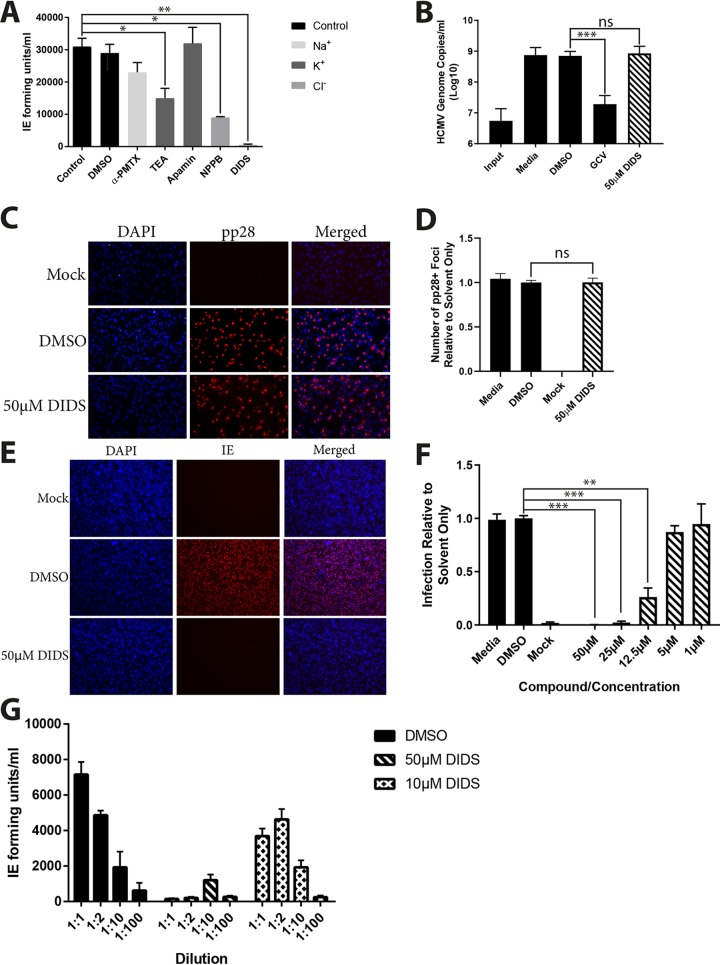
DIDS inhibits HCMV prior to IE gene expression. (A) HFFs were infected at an MOI of 5 and treated with ion channel inhibitors 24 hpi. Five days postinfection (dpi), the supernatants were used to inoculate fresh HFF monolayers, which were immunostained for viral IE expression 24 h later. Infection was assessed by automated fluorescence microscopy, and IE-forming units per milliliter were calculated (*n* = 3). (B) HFFs were infected at an MOI of 3; 24 hpi, cells were either harvested for DNA (Input) or treated with the indicated compounds. At 96 hpi, DNA was harvested under all conditions, and HCMV genome copies per milliliter were assessed by qPCR (*n* = 2). (C and D) HFFs were infected at an MOI of 3 and treated with the indicated compounds 24 hpi. Three days postinfection (p.i.), the cells were fixed and stained for viral pp28 expression (C), and pp28^+^ foci were counted manually by fluorescence microscopy (*n* = 2) (D). (E and F) HFFs were treated for 1 h at a range of DIDS concentrations or with controls prior to infection at an MOI of 3. The cells were fixed 24 hpi, stained for viral IE (E), and counted by automated fluorescence microscopy (*n* = 3) (F). (G) HFFs infected at an MOI of 3 were treated with DMSO, 50 μM DIDS, or 10 μM DIDS 24 hpi. Five days p.i., the supernatants were used to infect fresh HFF monolayers at varying dilutions, which were fixed 24 hpi and immunostained for IE proteins, and IE-forming units per milliliter were calculated as described above (*n* = 2). *P* values were calculated by a Kruskal-Wallis test with Dunn’s multiple-comparison test where appropriate. ***, *P* < 0.001; **, *P* < 0.01; *, *P* < 0.05; ns (nonsignificant), *P* > 0.05. The error bars represent standard deviations.

**FIG 2:**
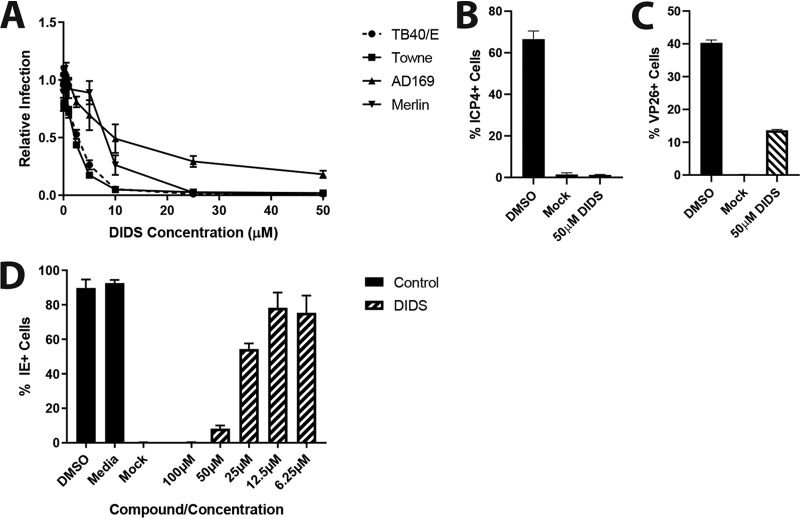
DIDS inhibits multiple HCMV strains, in addition to HSV-1. (A) HFFs were treated for 1 h at a range of DIDS concentrations or with controls prior to infection at an MOI of 3 with the Merlin, RC256 (Towne), AD169, or TB40/E-UL32-GFP strain of HCMV. The cells were fixed 24 hpi, stained for viral IE, and counted by automated fluorescence microscopy (*n* = 3). (B and C) HFFs were treated for 1 h with 50 μM DIDS or DMSO prior to infection at an MOI of 1 with HSV-1-VP26-GFP. The cells were fixed 4 hpi and stained for ICP4 expression, and ICP4^+^ cells were quantified (*n* = 1) (B), or fixed 24 hpi and VP26-GFP^+^ cells were quantified (*n* = 1) (C). (D) 3T3 cells were treated for 1 h at a range of DIDS concentrations or with controls prior to infection at an MOI of 3 with MCMV strain Smith in the absence of serum. The cells were washed 1 hpi, and the DIDS-containing medium was refreshed. The cells were fixed for 24 h, stained for viral IE proteins, and counted by automated fluorescence microscopy (*n* = 1). The error bars represent standard deviations.

On the initiation of these studies, our original remit was to test whether cellular ion channel activity played an important role in HCMV replication. A small-scale analysis of broadly acting ion channel inhibitors in a single-round infection assay revealed that one compound, DIDS, was a potent inhibitor of HCMV replication when added 6 h postinfection (hpi) (Fig. 1A). To identify the basis for the DIDS antiviral effect, we analyzed different aspects of the HCMV life cycle. Surprisingly, this revealed that DIDS was not inhibitory to viral DNA replication or the formation of putative virion assembly compartments (Fig. 1B and C). Thus, by these analyses, the viral life cycle appeared normal, yet infectious-virion production appeared to be impaired. Serendipitously, a time-of-addition study revealed that the pretreatment of cells with DIDS prior to infection potently inhibited viral infection at the level of initiation of immediate-early (IE) gene expression (Fig. 1D and E). Thus, we considered whether a potential explanation for the apparent lack of infectivity observed in the supernatants analyzed in the original screen (Fig. 1A) could be the persistence of DIDS in the culture media in the original assays impairing our assay’s ability to measure the presence of infectious virus in our supernatants. Consistent with this, dilution of the supernatants from the DIDS-treated cells (tested as shown in Fig. 1A) revealed the presence of infectious virus (Fig. 1F), suggesting that the original effect of DIDS observed was not an impact on replication *per se* but long-term persistence of the inhibitor in the media impacting the ability of the replicated virus to infect new target cells (which was our surrogate measure of viral replication). We went on to confirm that DIDS was capable of inhibiting multiple strains of HCMV at the level of IE gene expression, with the AD169, Towne (RC256), and TB40/E-UL32-GFP (green fluorescent protein) strains all strongly inhibited by DIDS (Fig. 2A). Additionally, this inhibition was not specific to HCMV, with herpes simplex virus (HSV)-VP26-GFP infection of human fetal foreskin fibroblasts (HFFs) powerfully inhibited by DIDS, whether by assessment of ICP4-positive (ICP4^+^) cells 4 hpi (Fig. 2B) or VP26-GFP^+^ cells 24 hpi (Fig. 2C). DIDS additionally inhibited the infection of murine cells by murine cytomegalovirus (MCMV), as quantified 24 hpi by staining for IE proteins (Fig. 2D).

**FIG 3:**
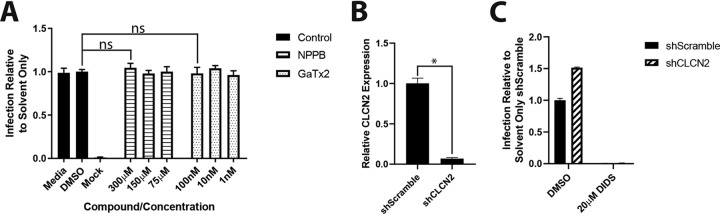
Alternative chloride ion channel inhibitors or knockdown of the major target of DIDS does not inhibit HCMV infection. (A) HFFs were treated with NPPB or GaTx2 at a range of concentrations for 1 h prior to infection at an MOI of 3. After 24 h, the cells were fixed and immunostained for viral IE proteins, and infection was quantified as previously described (*n* = 3). (B) Levels of CLCN2 RNA in HFFs transduced with shScramble- or shCLCN2-expressing lentiviral vectors were assessed by qRT-PCR. (C) shScramble- or shCLCN2-transduced HFFs were treated with DMSO or 20 μM DIDS for 1 h prior to infection at an MOI of 3. Infection was assessed by IE immunostaining 24 hpi as previously described (*n* = 2). *P* values were calculated by a Kruskal-Wallis test with Dunn’s multiple-comparison test or Mann-Whitney test where appropriate. *, *P* < 0.05; ns, *P* > 0.05. The error bars represent standard deviations.

Having established that DIDS was most active against the early stages of viral entry and the initiation of viral IE gene expression, we next sought to understand why it was inhibitory. DIDS is a well-established chloride ion channel inhibitor, and thus, we tested the abilities of other known chloride channel inhibitors to prevent HCMV infection. Unlike DIDS, 5-nitro-2-(3-phenylpropylamino)benzoic acid (NPPB) and GaTx2 had no impact on infection in the entry assay (Fig. 3A). Furthermore, depletion of the canonical target of DIDS (CIC-2, encoded by the *clcn2* gene) from cells by short hairpin RNA (shRNA) (validated as shown in Fig. 3B) was similarly not inhibitory to HCMV infection. Moreover, the infection of cells depleted of CIC-2 was still prevented by DIDS (Fig. 3C).

**FIG 4:**
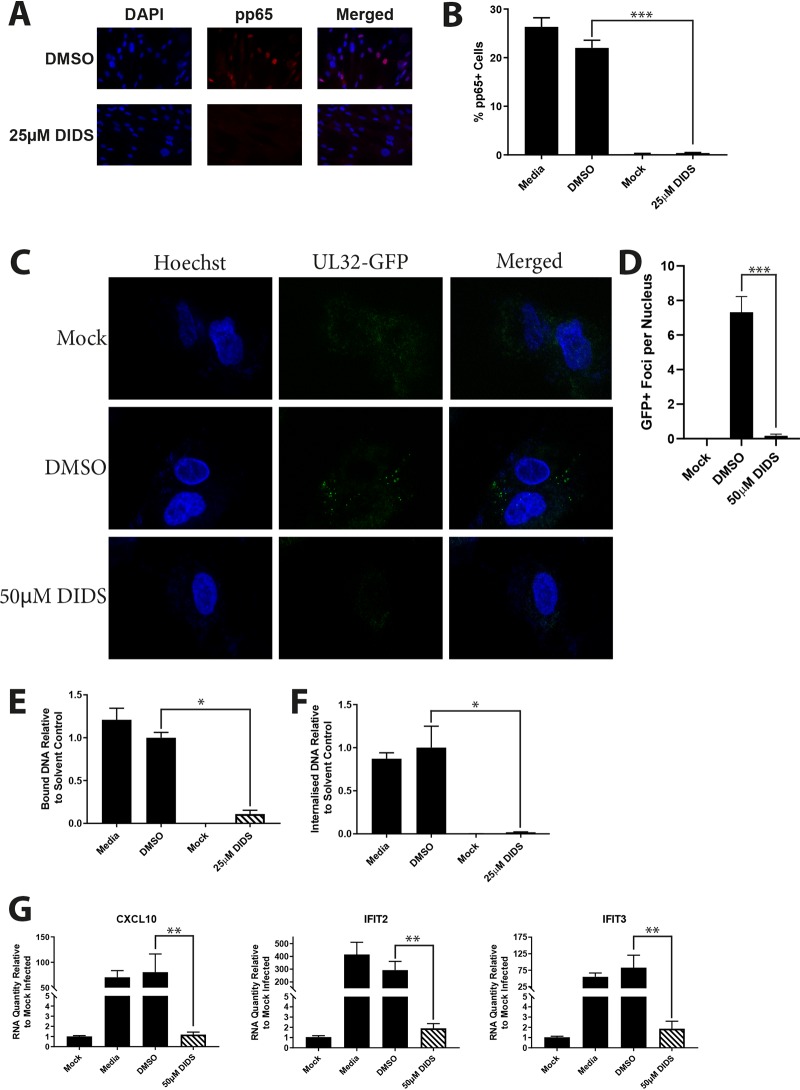
DIDS prevents engagement of HCMV with the cell surface and the infection of multiple cell types. (A and B) HFFs were treated with the indicated compounds prior to infection at an MOI of 2; 30 min postinfection, the cells were fixed and immunostained for viral pp65. Representative images are shown in panel A and enumeration by automated fluorescence microscopy in panel B (*n* = 2). (C and D) HFFs prelabeled with Hoechst were infected at an MOI of 5 with TB40/E-UL32-GFP in the presence of 50 μM DIDS for 1 h at 4°C. The cells were then washed and visualized by confocal microscopy. Representative images (C) and quantification (D) are provided. (E and F) HFFs were infected at an MOI of 2 at 4°C for 1 h in the presence of the indicated compounds. The cells were then washed and DNA extracted immediately (E), or the cells were returned to 37°C for 30 min prior to DNA extraction (F). Relative levels of HCMV DNA were assessed by qPCR (*n* = 3). (G) HFFs were infected at an MOI of 2 in the presence of the indicated compounds. Six hours p.i., RNA was extracted, and induction of CXCL10, IFIT2, and IFIT3 was assessed by qRT-PCR (*n* = 3). *P* values were calculated by a Kruskal-Wallis test with Dunn’s multiple-comparison test or Mann-Whitney test where appropriate. ***, *P* < 0.001; **, *P* < 0.01; *, *P* < 0.05. The error bars represent standard deviations.

**FIG 5:**
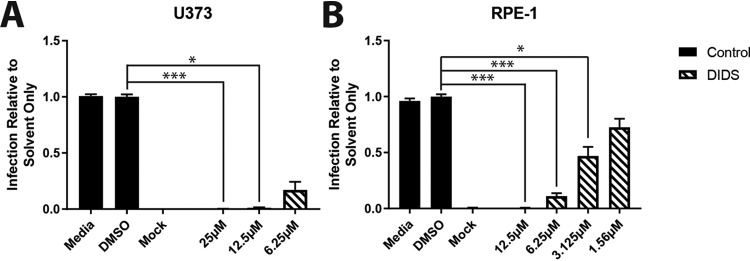
DIDS inhibits the infection of multiple cell types by HCMV. (A and B) U373 cells (A) or RPE-1 cells (B) were pretreated for 1 h with a range of concentrations of DIDS or controls prior to infection at an MOI of 3; 24 hpi, the cells were fixed, and infection was assessed by IE immunostaining as previously described (*n* = 3). ***, *P* < 0.001; *, *P* < 0.05. The error bars represent standard deviations.

The data pointed toward an event prior to IE gene expression that was targeted by DIDS activity and possibly independent of ion channel activity. To begin identification of the stage of the viral entry pathway impacted by DIDS, we analyzed the delivery of virion components to the cells (Fig. 4). Immunofluorescence analysis revealed that pp65 tegument failed to translocate to the nucleus in the presence of DIDS, demonstrating that an event prior to IE expression was indeed being targeted by DIDS (Fig. 4A and B). Furthermore, fluorescently labeled viruses failed to bind to the surfaces of the cells in the presence of DIDS (Fig. 4C and D) during infection at 4°C, which otherwise allows binding of the virus to the plasma membrane, but not efficient entry into the cell. The microscopy data were augmented by an assessment of binding and internalization of virions by detection of viral DNA by quantitative PCR (qPCR), which revealed that not only was viral DNA not internalized into the cell following the promotion of viral entry by warming to 37°C after infection at 4°C in the presence of DIDS (Fig. 4E), but detection of viral DNA bound to the cell surface was greatly reduced by DIDS (Fig. 4F), reinforcing the evidence that DIDS prevents the virus from binding to the cell surface. In support of limited contact between the virion and the plasma membrane was the observation that HCMV induction of interferon-stimulated-gene expression was absent in cells infected in the presence of DIDS (Fig. 4G). Taken together, these data pointed to an event during the very early stages of viral entry, possibly during the initial stages of virus binding. Furthermore, DIDS inhibited the infection by HCMV of multiple cell types, arguing that the activity of DIDS was directed against a common event in the entry process (Fig. 5A and B).

**FIG 6:**
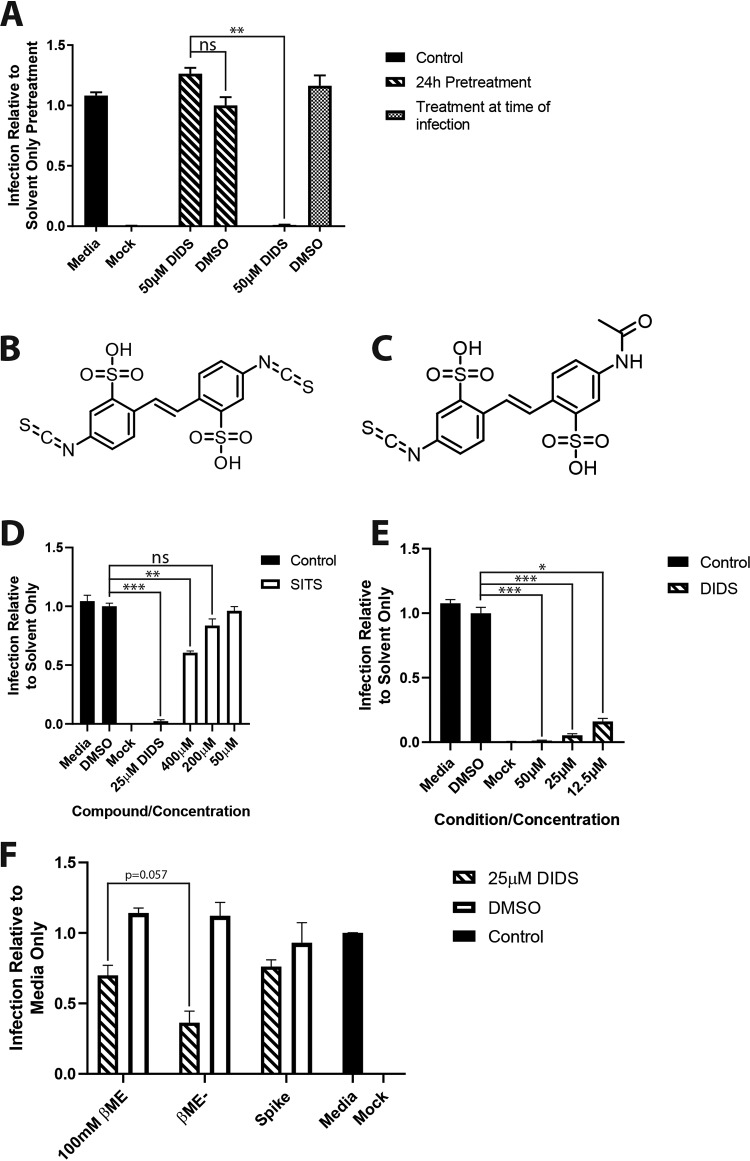
DIDS inhibits HCMV infection by targeting the virion. (A) HFFs were either untreated or treated with DMSO or 50 μM DIDS for 24 h. The compound was removed from the pretreated cells, the untreated cells had either 50 μM DIDS or DMSO added, and all the cells were infected at an MOI of 2 for 1 h. The cells were then washed, and medium only was added to all the cells. Infection was quantified by IE immunostaining 24 hpi as described previously (*n* = 2). (B) Structure of DIDS (4,4′-diisothiocyano-2,2′-stilbenedisulfonic acid). (C) Structure of SITS (4-acetamido-4′-isothiocyanato-2,2′-stilbenedisulfonic acid). (D) HFFs were treated with 25 μM DIDS or a range of concentrations of SITS for 1 h prior to infection at an MOI of 2. Infection was quantified by IE immunostaining 24 hpi as described previously (*n* = 3). (E) HCMV was treated with the indicated compounds for 1 h. Samples were then pelleted by centrifugation, resuspended in medium only, and used to inoculate the HFFs. Eight hours p.i., the cells were immunostained for IE proteins, and infection was quantified as described previously (*n* = 3). (F) Virus was treated with 25 μM DIDS, DMSO, or medium only for 1 h. The DIDS/DMSO-treated virus was then either treated with 100 mM βME or left untreated (βME–) for 10 min. All the preparations were then diluted, and previously untreated virus had sufficient DIDS or DMSO added (Spike) to create the same final concentration present in the samples pretreated with either DIDS or DMSO. The virus was then used to inoculate HFFs at an MOI of 1, with cells stained 24 hpi and infection quantified as described previously (*n* = 2). *P* values were calculated by a Kruskal-Wallis test with Dunn’s multiple-comparison test or Mann-Whitney test where appropriate. ***, *P* < 0.001; **, *P* < 0.01; *, *P* < 0.05; ns, *P* > 0.05. The error bars represent standard deviations.

An inherent assumption of the study thus far was that DIDS was inhibiting the activity of a cellular function required for entry. However, if cells were pretreated with DIDS for 24 h but then washed and infected for 1 h in the absence of DIDS, DIDS’ inhibitory activity on HCMV infection was abolished (Fig. 6A). Two explanations were plausible: the inhibition was rapidly reversible, or the target was virus encoded. We noted that the DIDS molecule carries a negative charge, and thus, clear parallels with heparin—a known inhibitor of HCMV entry—were evident. To investigate the impact of the charge, we took the charged molecule 4-acetamido-4′-isothiocyanato-2,2′-stilbenedisulfonic acid (SITS), which is closely related to DIDS (Fig. 6B and C) except that one isothiocyanate-reactive group is exchanged for an acetamide group, and asked if it inhibited HCMV (Fig. 6D). Even an extremely high concentration of SITS (∼45 times greater than the 50% inhibitory concentration [IC_50_] of DIDS) had only a minor effect on HCMV infection, rendering it far less potent than DIDS, arguing that the basis of DIDS inhibition was not the charge alone. Furthermore, it suggested that the reactivity of the isothiocyanate groups may be responsible, as loss of the group represented the only difference between the two molecules. Biochemical studies have suggested that the presence of dual isothiocyanate groups would render DIDS reactive with lysine and cysteine, whereas SITS displays preferential activity toward lysine residues (44). Thus, we hypothesized that the reactive isothiocyanate groups may promote the interaction of DIDS with the virus via cysteine interactions. To test for a virus-DIDS interaction, we incubated HCMV with DIDS prior to infection for 1 h. The virus was then purified by centrifugation, resuspended in fresh DIDS-free medium, and used to infect cells. The data show that the entry of HCMV was still dramatically reduced (Fig. 6E). Consistent with cysteine reactivity underpinning the impact of DIDS on the virus, we observed that treatment of the DIDS-virus mixture with the reducing agent β-mercaptoethanol (βME) could partially reverse the effects of DIDS and restore the infectivity of the DIDS-treated virus (Fig. 6F). Furthermore, the ability of β-mercaptoethanol to reverse the effects of DIDS also demonstrated that DIDS was not antiviral due to toxicity against the virion. The virions clearly remained viable, as they were infectious if the interaction with DIDS was reversed.

**FIG 7:**
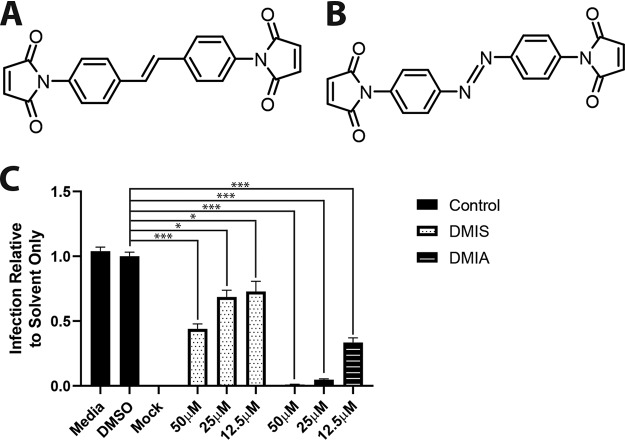
Cysteine-reactive compounds structurally related to DIDS inhibit HCMV. (A) Structure of DMIS (4,4′-dimaleimidylstilbene). (B) Structure of DMIA (4,4′-dimaleimidylazobenzene). (C) HCMV was treated with the indicated compounds for 1 h. Samples were then pelleted by centrifugation, resuspended in medium only, and used to inoculate the HFFs. Twenty-four hours p.i., the cells were immunostained for IE proteins, and infection was quantified as described previously. *P* values were calculated by a Kruskal-Wallis test with Dunn’s multiple-comparison test. ***, *P* < 0.001; *, *P* < 0.05. The error bars represent standard deviations.

**FIG 8:**
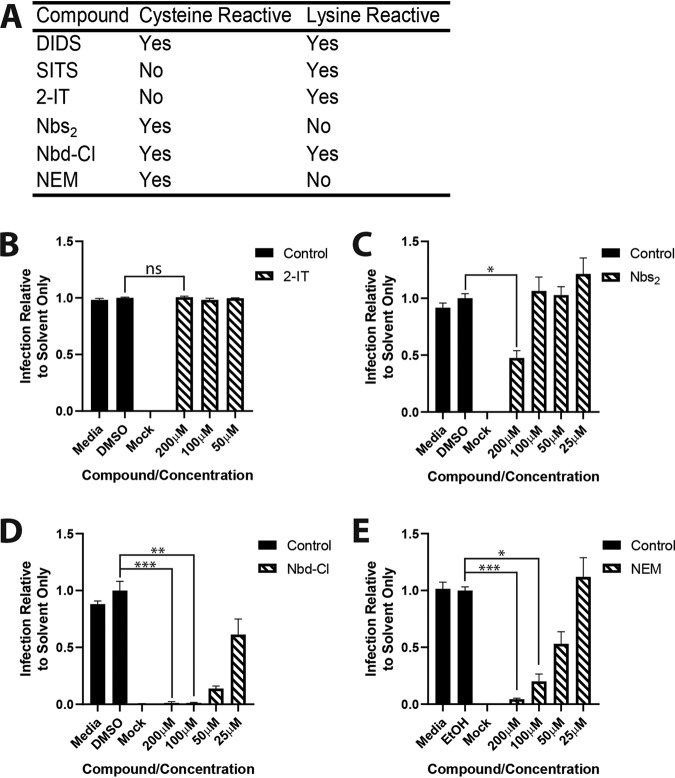
HCMV infection is sensitive to the activity of structurally unrelated cysteine-reactive compounds. (A) Summary of reactivities of tested compounds. (B to E) HCMV was treated with the indicated compounds (2-IT [B], Nbs_2_ [C], Nbd-Cl [D], or NEM [E]) for 1 h. The virus was then pelleted, resuspended in medium only, and used to inoculate HFFs. Twenty-four hours p.i., the cells were immunostained for IE proteins, and infection was enumerated as described previously. *P* values were calculated by a Kruskal-Wallis test with Dunn’s multiple-comparison test where appropriate. ***, *P* < 0.001; **, *P* < 0.01; *, *P* < 0.05; ns, *P* > 0.05.

Thus, the data pointed toward cysteine reactivity in the DIDS molecule being important. The ideal experiment was to generate a version of DIDS with charged sulfonated groups removed. However, the resultant compound proved highly insoluble. An alternative approach used the exchange of the isothiocyanate groups with similarly cysteine-reactive maleimide groups, which allowed the generation of noncharged isothiocyanate-like derivatives (Fig. 7A and B). Both of the compounds remained inhibitory to HCMV infection, although 4,4′-dimaleimidylazobenzene (DMIA) appeared to display better potency than 4,4′-dimaleimidylstilbene (DMIS) (Fig. 7C). Further support for cysteine reactivity being antiviral was established through the testing of a panel of compounds previously characterized for cysteine and lysine reactivity (Fig. 8A). The data show that compounds with cysteine reactivity inhibited HCMV infection under the same experimental conditions as those observed for DIDS (Fig. 8B to E). It was also noteworthy that compounds with dual reactivity (i.e., DIDS and 4-chloro-7-nitrobenzofurazan [Nbd-Cl]) appeared to be more potent against HCMV than solely cysteine-reactive compounds.

**FIG 9:**
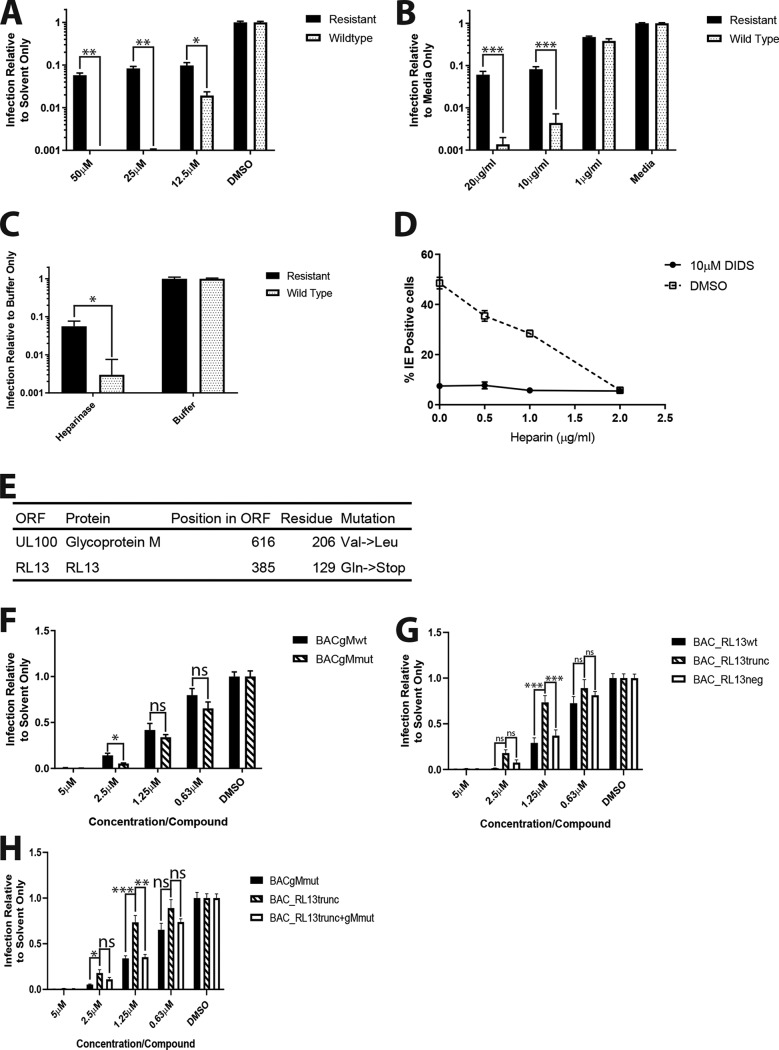
Virus passaged in the presence of DIDS additionally develops resistance to heparin and cellular heparan sulfate depletion. (A and B) DIDS-passaged HCMV (Resistant) and the virus from which it was derived (Wildtype) were used to infect HFFs at an MOI of 2 in the presence of either DIDS (A) or heparin (B). The cells were immunostained for IE 24 hpi, and infection was enumerated as described previously (*n* = 3). (C) HFFs were treated with heparinase I or buffer only for 1 h at 37°C. The cells were then infected at an MOI of 2 with resistant or wild-type HCMV, followed by IE immunostaining 24 hpi, and infection was enumerated as described previously (*n* = 2). (D) HFFs were infected with resistant HCMV at an MOI of 2 in the presence of increasing concentrations of heparin, in addition to either 10 μM DIDS or DMSO. Infection was quantified 24 hpi by IE immunostaining as described previously (*n* = 2). (E) Summary of mutations found to be enriched within DIDS-resistant HCMV by sequencing. ORF, open reading frame. (F to H) BAC-derived viruses lacking RL13 with wild-type gM (BACgMwt), lacking RL13 with mutant gM (BACgMmut), possessing wild-type gM with either wild-type RL13 (BAC_RL13wt) or truncated RL13 (BAC_RL13trunc) or lacking RL13 (BAC_RL13neg), or possessing both truncated RL13 and mutant gM (BAC_RL13trunc+gMmut) were used to infect HFFs at an MOI of 1 in the presence of DIDS. After 24 h, the cells were fixed and immunostained for viral IE proteins, and infection was quantified as described previously (*n* = 3). *P* values were calculated by a Mann-Whitney or Kruskal-Wallis test with Dunn’s multiple-comparison test where appropriate. ***, *P* < 0.001; **, *P* < 0.01; *, *P* < 0.05; ns, *P* > 0.05. The error bars represent standard deviations.

To try to derive some biological insight from these studies of DIDS, a DIDS-resistant strain was generated with a view to the identification of a viral target for DIDS. Wild-type strain Merlin (NC_006273.2) was passaged in the presence of increasing concentrations of DIDS to isolate a resistant strain. After 6 months, a mixed population of Merlin viruses containing a highly DIDS-resistant fraction was produced (Fig. 9A). Interestingly, the DIDS-resistant virus population was also resistant to the inhibitory effects of heparin (Fig. 9B) and could infect heparinase-treated cells (Fig. 9C), suggesting that the generation of DIDS resistance had altered the entry pathway by circumventing the initial steps of viral entry and HSPG dependence. It was clear that the infectivity of the resistant virus never reached 100% in the presence of DIDS or heparin, and thus, the heparin and DIDS sensitivities could conceivably be due to different mutants in the virus stock. However, when DIDS and heparin were used in combination, no additive impact on infection with the DIDS-resistant virus was observed, suggesting that the same fraction was resistant to both (Fig. 9D).

The resistant population of HCMV was sequenced and revealed enrichment for two mutations: a change from valine to leucine at position 206 in UL100 (gM) and enrichment for a population containing a premature stop codon at residue 129 in RL13 in comparison to the original wild-type virus, which contained a raft of different RL13 mutations (Fig. 9E). Given the role of gM in entry and, particularly, events associated with HSPGs, this was investigated first. However, the introduction of the gM mutation into a Merlin BAC lacking RL13 had no impact on the sensitivity of the virus to DIDS (Fig. 9F). In contrast, introduction of the mutation that generated the truncation in RL13 identified in the sequencing did provide partial evidence of a resistance phenotype, although only a minor impact was observed in comparison to the original DIDS-resistant virus (Fig. 9G). However, it was intriguing that both RL13^+^ and RL13-negative (RL13^−^) viruses were similarly sensitive to DIDS, whereas the RL13 truncation did produce slightly more resistance to the effects of DIDS (Fig. 9F) at specific DIDS concentrations, suggesting the specific expression of the truncated form of RL13 may play a role. Finally, we generated the double mutant, but again, no overt phenotype was observed that would explain the DIDS resistance of the original DIDS-resistant strain (Fig. 9H). However, we were intrigued to note that any benefit regarding resistance to DIDS proffered by the RL13 truncation was abrogated when the gM mutation was introduced concomitantly (Fig. 9H).

**FIG 10:**
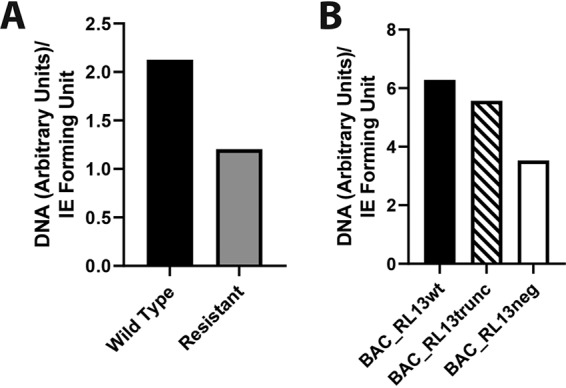
DIDS-resistant viruses display a moderate increase in infectivity. HFFs were infected with serial dilutions of wild-type and DIDS-resistant HCMV (A) or BAC_RL13wt, BAC_RL13trunc, and BAC_RL13neg (B) in the presence of medium only. Simultaneously, samples of virus were pelleted by centrifugation, and DNA was extracted. The HFFs were immunostained at 24 hpi for IE proteins, infection was enumerated, and IE-forming units per milliliter were calculated for each virus. Quantitative PCR for viral genomes was performed on the viral DNA samples, and DNA per milliliter was calculated. DNA per milliliter was then divided by IE-forming units per milliliter to produce a genomes/infectious-unit ratio for each virus.

Given that the genetic mutants made in the Merlin BAC viruses did not map the generation of DIDS-resistant virus to a specific gene, we tested the hypothesis that the virus could acquire resistance to DIDS by altering the particle/infectious-virus ratio. The rationale was that the provision of more targets for DIDS to bind to might lower the effective DIDS concentration available to inhibit the infectious particles. Consequently, we compared the DNA content/infectious-virus ratio for the DIDS-resistant virus to that of the wild type, as well as comparing the ratios for BAC_RL13wt, BAC_RL13trunc, and BAC_RL13neg (see below). Although no dramatic differences in the ratios were observed, we note that the data suggest that the DIDS-resistant virus displayed an approximately 2-fold increase in infectivity (Fig. 10A). In contrast, in the BAC mutants, the ratios did not show any consistent variation that correlated with DIDS resistance (Fig. 10B).

**FIG 11:**
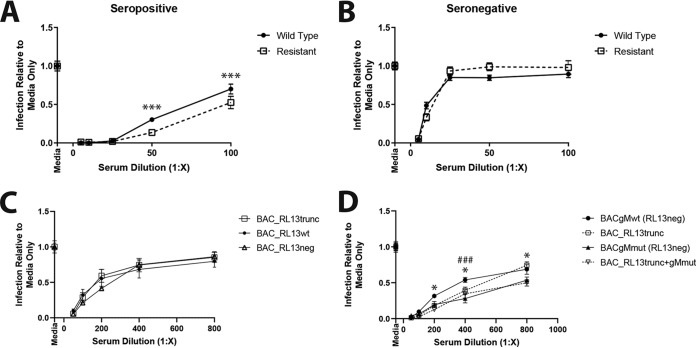
DIDS-resistant virus is sensitive to humoral immunity. (A and B) DIDS-resistant or wild-type HCMV was treated with the indicated dilution of either pooled seropositive (A) or pooled seronegative (B) serum for 1 h. The treated virus was then used to infect HFFs at an MOI of 1, the cells were immunostained 24 hpi for IE proteins, and infection was enumerated as described previously (*n* = 2). ***, *P* < 0.001. (C and D) BAC-derived viruses expressing variations of RL13 (C) or the RL13 truncation and gM mutation in all combinations (D) were treated with the indicated dilutions of pooled seropositive serum for 1 h. The treated virus was used to infect HFFs at an MOI of 1, the cells were immunostained 24 hpi for IE proteins, and infection was enumerated as described previously (*n* = 3). *P* values were calculated by 2-way analysis of variance (ANOVA) with Tukey’s multiple-comparison test. ###, *P* < 0.001 for BACgMwt versus BACgMmut; *, *P* < 0.05 for BACgMwt versus BAC_RL13trunc+gMmut. The error bars represent standard deviations.

Although the genetic basis of the resistance was not unequivocally revealed, we were intrigued by the observation that HCMV could be forced by DIDS to mutate *in vitro* to enrich for a fraction of virions that are HSPG independent for entry. This led us to consider the molecular and evolutionary bases for the retention of this interaction in wild-type HCMV strains *in vitro* and, more pertinently, *in vivo*. Complexity in the entry pathways of a number of viruses has been hypothesized to provide a mechanism of immune evasion. Essentially, there is an evolutionary trade-off between efficient and rapid delivery of the viral cargo and the effective evasion of humoral immune responses. Consequently, the concept of conformational masking, where the virus exposes only key epitopes required for entry at a specific stage of the process, has developed. The key tenet is that conserved epitopes essential for entry cannot tolerate extensive mutation as a mechanism to evade immune surveillance. These epitopes are instead hidden from the immune response for as long as possible to limit the opportunity for immune recognition. Thus, we wished to test whether resistance to DIDS, and by extension heparin, was met with a selective disadvantage for the virus via failure to evade humoral immunity. Both wild-type and DIDS-resistant viruses were incubated with pooled sera from seropositive or seronegative donors, and then infectivity was measured. The data show that, while both wild-type and resistant viruses are sensitive to antibody neutralization, titration of seropositive sera revealed increased sensitivity of the original DIDS-resistant virus to neutralization at higher dilutions of sera (Fig. 11A and B). Next, the sensitivity of the mutant BAC viruses was tested to identify whether increased sensitivity could be explained by RL13 or gM (Fig. 11C and D). Here, the BAC-generated mutants presented a complicated phenotype. The expression of the gM mutant in a RL13-null background rendered the virus more sensitive to neutralization (Fig. 11D). The introduction and expression of the RL13 truncation did not have a consistent effect on the antibody sensitivity phenotype of the wild-type virus (Fig. 11C) and did not additionally contribute the gM phenotype (Fig. 11D).

**FIG 12:**
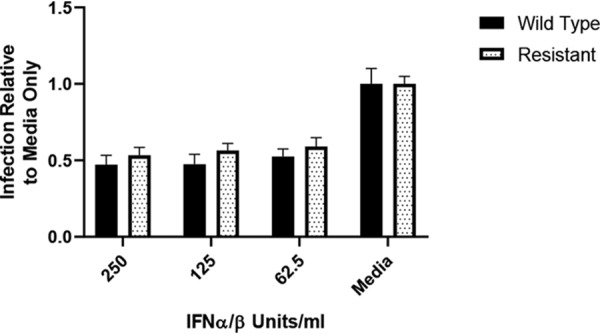
DIDS-resistant virus is not sensitized to antiviral type I interferon activity. HFFs were treated for 3 h with the indicated quantity of IFN-α/β prior to infection at an MOI of 1. Twenty-four hours p.i., the cells were fixed and immunostained for IE, and infection was quantified as escribed previously (*n* = 2). The error bars represent standard deviations.

To test the hypothesis that the DIDS-resistant virus was intrinsically more sensitive to immune responses, we asked whether the DIDS-resistant virus was rendered generally more susceptible to additional immune activity during the early phases of viral infection. However, in contrast to the studies with humoral immunity, the wild-type and DIDS-resistant viruses were equally sensitive to the antiviral activity of type I interferons (IFNs) in IFN-pretreated HFFs (Fig. 12).

## DISCUSSION

HCMV entry is a complex process that requires a concert of viral glycoprotein-cell surface interactions to occur. Although the list of specific receptors that facilitate HCMV entry continues to grow, the initial interaction of HCMV with HSPGs is a well-established interaction important for viral entry (2). This initial binding via HSPGs is a common event for the entry processes of many viruses and has long been considered a mechanism to promote colocalization of the virion with the plasma membrane, which potentially could enhance infectivity (16–18). Thus, an antiviral strategy that prevents this initial step represents an attractive strategy, particularly given the role of this interaction in the infectivity of many enveloped viruses. However, it is less well understood whether the virus garners any additional benefits from this interaction beyond the concept that HSPGs provide a means to promote initial virus engagement with the cell and thus could mutate rapidly into an HSPG-independent strain of virus as a mechanism to evade the activity of antivirals.

Here, we report that DIDS is inhibitory to HCMV infection through a blockade of the ability of the virus to bind and engage with the plasma membrane. From a translational standpoint, inhibitors of viral entry have long represented an attractive focus for new antivirals. Although DIDS carries a negative charge, it became clear that cysteine reactivity played in an important role in its antiviral activity. Indeed, a number of cysteine-reactive compounds appeared to inhibit HCMV infection. A simple explanation could be that the binding of DIDS (and other cysteine-reactive compounds) to virions provides steric hindrance. In that case, it is perhaps surprising that lysine-reactive compounds cannot achieve the same effect, since viral glycoproteins contain multiple lysine residues. The relative accessibility of the lysines versus cysteines may explain the difference, although lysine is an amphipathic amino acid and thus can be found buried within the protein structure and simultaneously can decorate the protein surface (45, 46).

Studies of hepatitis delta virus (HDV), which incorporates HBV glycoproteins in the virion, have shown that cysteine residues play an important role in infection due to conformational changes they promote via an ability to catalyze thiol-disulfide exchange (47). As such, compounds that prevented thiol-disulfide activity blocked HDV infection. A number of HCMV glycoproteins contain substantial numbers of cysteines, and thus, this may invoke a model whereby DIDS decorates the virion surface and possibly prevents similar cysteine-driven activities. HCMV glycoproteins have been shown to undergo conformational changes during the entry process, and cysteine residue activity possibly plays a role in this, rendering cysteine-reactive compounds more potent as antivirals (30).

However, any cysteine-targeted activity does not rule out the possibility that the charge associated with DIDS could also play a role in augmenting its activity against HCMV, especially since charge is considered the basis of the antiviral activity of heparin against HCMV. Binding of DIDS to the virus may introduce a localized negative charge that contributes to the inhibition of infection. Furthermore, it is likely that the charge carried by the DIDS molecule is responsible for the lack of cellular and *in vivo* toxicity observed with DIDS (48). It was evident in cultures that a number of cysteine-reactive derivatives that did not carry a charge were far more toxic than DIDS, despite the fact that DIDS also carries highly reactive isothiocyanate groups. We hypothesize that DIDS is poor at crossing cell membranes due to the presence of the charged sulfonated groups. This prediction is borne out by the original screen, which suggested that DIDS persisted in the extracellular medium for 5 days, as evidenced by its ability to diminish the infectivity of newly released viruses after a single round of replication. Of course, inability to cross membranes comes with a cost for any potential therapeutic derivatives of DIDS, as their delivery would likely require strategies that facilitate transport across the epithelial cell membranes if administered orally, for example. Furthermore, it may limit the impact of DIDS-like compounds against cell-associated HCMV if DIDS enters the cell inefficiently. On the other hand, a large difference between 50% cytotoxic concentration (CC_50_) and 50% effective concentration (EC_50_) values provides a good therapeutic index, which is always a desirable property in any pharmacological intervention, particularly when transitioning from cell culture to *in vivo* models.

It was disappointing that a single mutation to fully explain the resistance phenotype was not evident. The RL13 truncation had a partial phenotype but did not fully recapitulate the original DIDS-resistant virus phenotype. It is important to note that the partial phenotype might have been influenced by the requirement to grow RL13trunc- or RL13wt-expressing viruses in HFFs in order to allow the expression of RL13. One potential issue is the backbone of the viruses used. The original resistant virus was generated using the wild-type strain Merlin, which had already been passaged in the laboratory and is likely more heterogeneous as a virus stock. In contrast, the RL13 truncation was generated in the BAC Merlin, which is likely to be genetically more homogeneous than the original Merlin virus used. For example, the RL13 phenotype may be enhanced by minor mutations accumulating in the Merlin virus through culture *in vitro*, and thus, resistance via RL13 is enhanced by other, as yet unidentified polymorphisms. Essentially, the accumulation of the RL13 and gM mutations in the original resistant virus suggests they are important for resistance, but their impact is underpinned by fixed mutations present in our passaged Merlin stocks that have been acquired over time and are absent from the BAC virus (which has been repaired to reflect the original consensus sequence derived from clinical studies). Certainly, the data do suggest that the BAC-derived viruses are intrinsically more sensitive to DIDS, with inhibition of infection seen at much lower concentrations of DIDS than with non-BAC-derived Merlin stocks. This argues that the original Merlin carries fixed single nucleotide polymorphisms (SNPs) that contribute to the resistance phenotype. It also raises a tangential but important question when considering the potential potency of new therapeutics. Specifically, how diverse are HCMV populations *in vivo* and, consequently, is the relative genetic homogeneity associated with BAC viruses completely reflective of the *in vivo* situation and do they potentially give unfair or even unrepresentative measures of potency *in vitro*?

An important aspect of the pharmacological data is that the EC_50_s for both resistant and wild-type viruses were very similar. The reason for this is that it was a fraction within the DIDS-resistant virus that was responsible for the resistance rather than an overall increase in the resistance of the HCMV strains to DIDS. This supports the concept that a single mutant identified in >90% of viruses cannot recapitulate the phenotype and partially explains the lack of phenotype in the reengineered RL13 and gM viruses. Indeed, we revisited the sequencing data to identify whether there was evidence of enrichment for another genetic mutation by around 10 to 15%, but this was not evident. Thus, we hypothesize that this may point to the acquisition of multiple mutations alongside predetermined SNPs that generate the DIDS-resistant subpopulation that accumulates in the culture. Indeed, attempts to expand the frequency of resistance in the DIDS-resistant virus have not resulted in the accumulation of a higher frequency of DIDS-resistant viruses.

Finally, a serendipitous aspect of the data was the suggestion that HCMV can be driven to mutate to infect cells in an HSPG-independent manner, yet to date, all the wild-type strains of HCMV tested utilize HSPG binding during the entry process. Thus, the retention of this activity argues that, from an evolutionary aspect, HSPG interactions are indeed important. One hypothesis we tested was whether increased complexity in the entry process via engagement with multiple receptors may promote reduced recognition by the humoral immune response. This idea, termed conformational masking, is not unique to HCMV and was established through studies of HIV, where the virus engages with CD4 before secondary interaction with CCR5 or CXCR4, with the important consequence of reducing antibody recognition and neutralization of CXCR4/CCR5 binding epitopes of HIV gp120 (49). Although preliminary data suggested increased sensitivity to humoral immunity and it mapped functionally to a mutation in the HSPG binding protein, gM, whether this represents increased sensitivity to anti-gM antibodies or just changes in the virion proteome induced by the gM mutation alone is not clear. Paradoxically, this mutation alone did not explain the DIDS resistance, so it is not clear whether the two events (DIDS resistance and antibody sensitivity) are inherently linked. However, it is possible that, if the mutation is one of multiple events that contribute to DIDS resistance, then it would provide the link. Experimentally, repairing the gM mutation in the original DIDS-resistant virus would address this, but it first requires a complete understanding of the genetic basis of the DIDS phenotype.

In summary, we report novel antiviral activity of a putative chloride ion channel inhibitor active against HCMV during the initial stages of entry. DIDS’ inhibitory activity is dependent on a reversible cysteine reactivity driven by isothiocyanate chemical groups and not through the targeting of host chloride ion channel function. Intriguingly, identification of a DIDS-resistant virus revealed that HCMV can mutate to utilize an HSPG-independent mechanism of entry and results in a concomitant increase in sensitivity to antibody neutralization. However, it remains to be determined whether the genetic basis for DIDS resistance is responsible for the acquired antibody sensitivity. The data also illustrate that cysteine-reactive compounds have potential as novel antivirals through an ability to bind and inhibit HCMV virions to prevent binding and entry into target cells.

## MATERIALS AND METHODS

### Cells and virus propagation.

HFFs (SCRC-1041) and retinal pigment epithelial cells (RPE-1 and CRL-4000) were purchased from ATCC. U373 cells were a kind gift from John Sinclair, University of Cambridge. HFF-Tet cells were a kind gift from Richard Stanton, Cardiff University. Murine 3T3 fibroblasts were kindly provided by Ian Humphreys, Cardiff University. All the cells were grown in high-glucose Dulbecco’s modified Eagle medium (DMEM) (Gibco) supplemented with 10% fetal bovine serum (FBS) (ThermoFisher), 100 U/ml penicillin, and 100 μg/ml streptomycin. Merlin, TB40/E-UL32-GFP (a kind gift from Christian Sinzger), Towne, and AD169 stocks of HCMV were propagated in HFFs. HSV-VP26-GFP was a kind gift from Richard Milne. Stocks of murine CMV strain Smith were kindly provided by Ian Humphreys. All experiments were performed with Merlin HCMV unless otherwise stated. BAC recombinant viruses were propagated in HFF-Tet cells to prevent mutations in the RL13 (under the control of a Tet repressor in BAC_RL13wt, BAC_RL13trunc, and BAC_RL13trunc+gMmut and defective in BACgMwt, BACgMmut, and BAC_RL13neg) and UL128-131 (under the control of a Tet repressor in BACgMwt and BACgMmut and defective in all other BAC viruses) loci and thus retain a wild-type genotype and were maintained as the seed stocks (50). Prior to their use, the virus seed stocks encoding either wild-type or truncated RL13 were then amplified in HFFs to allow expression of their respective RL13s, while RL13-negative variants were generated from HFF-Tet cells. Supernatants from cells infected with BAC recombinant viruses were purified using sorbitol gradient (20% d-sorbitol, 50 mM Tris, pH 7.4, 1 mM MgCl_2_) centrifugation (65,000 × *g* for 90 min at 4°C) with an SW32Ti rotor (Beckman Coulter) in an Optima XE ultracentrifuge (Beckman Coulter).

### Inhibitors and chemicals.

NPPB, apamin, α-pompilidotoxin (α-PMTX), and tetraethylammonium (TEA) were gifts from Jamel Mankouri (University of Leeds). GaTx2 was purchased from Tocris, while DIDS, SITS, *N*-ethylmaleimide (NEM), 2-iminothiolane (2-IT), Nbd-Cl, 5,5′-dithiobis(2-nitrobenzoic acid) (Nbs_2_), and heparin were all purchased from Sigma-Aldrich and used at the concentrations described in the figures. The concentrations of NPPB and GaTx2 used were based on the concentrations previously established to be required for inhibition of chloride ion channels (51, 52). For βME experiments, HCMV was pretreated with DIDS or dimethyl sulfoxide (DMSO) for 1 h and then subsequently incubated with βME for 10 min, diluted, and used to infect HFFs by a standard procedure. For enzymatic removal of heparin sulfates, HFFs were treated with heparinase I (New England Biolabs) at 30°C for 1 h prior to infection with HCMV.

### Synthesis of DMIA.

4-[(E)-(4-aminophenyl)azo]aniline (200 mg; 0.94 mmol) was dissolved in dimethylformamide (DMF) (8 ml) in a flask wrapped in foil, and to this, maleic anhydride (214 mg; 2.07 mmol) dissolved in DMF (8 ml) was added portionwise. The reaction mixture gradually turned a dark black/orange and was stirred for 2 h. The reaction mixture was then filtered, and the solid was washed with tetrahydrofuran (THF) (20 ml) and DCM (100 ml) and dried under vacuum to give (Z)-4-[4-[(E)-[4-[[(Z)-3-carboxyprop-2-enoyl]amino]phenyl]azo]anilino]-4-oxo-but-2-enoic acid (251 mg; 66% yield).

A solution of (Z)-4-[4-[(E)-[4-[[(Z)-3-carboxyprop-2-enoyl]amino]phenyl]azo]anilino]-4-oxo-but-2-enoic acid (251 mg; 0.62 mmol) in acetic anhydride (10 ml) was heated to 95°C, and then sodium acetate (202 mg; 2.46 mmol) was added. The reaction mixture was heated at 95°C for 2 h and then allowed to cool to room temperature. The mixture was poured onto ice water, stirred for 30 min, and then neutralized with aqueous sodium hydrogen carbonate solution and filtered, and the solid was air dried to give 4,4′-dimaleimidylazobenzene (128 mg; 56% yield). ^1^H nuclear magnetic resonance (NMR) (DMSO-d6; 400 MHz) 8.04 (4H, d), 7.63 (4H, d), and 7.26 (2, 4H).

### Synthesis of DMIS.

4-[(E)-2-(4-aminophenyl)vinyl]aniline (200 mg; 0.95 mmol) was dissolved in DMF (8 ml) in a flask wrapped in foil, and to this, maleic anhydride (216 mg; 2.09 mmol) dissolved in DMF (8 ml) was added portionwise. The reaction mixture gradually turned a dark black/orange and was stirred for 2 h. The reaction mixture was then filtered, and the solid was washed with THF (20 ml) and DCM (100 ml) and dried under vacuum to give (Z)-4-[4-[(E)-2-[4-[[(Z)-3-carboxyprop-2-enoyl]amino]phenyl]vinyl]anilino]-4-oxo-but-2-enoic acid (250 mg; 65% yield).

A solution of (Z)-4-[4-[(E)-2-[4-[[(Z)-3-carboxyprop-2-enoyl]amino]phenyl]vinyl]anilino]-4-oxo-but-2-enoic acid (250 mg; 0.62 mmol) in acetic anhydride (10 ml) was heated to 95°C, and then sodium acetate (202 mg; 2.46 mmol) was added. The reaction mixture was heated at 95°C for 2h and then allowed to cool to room temperature. The mixture was poured onto ice, stirred for 30 min, neutralized with sodium hydrogen carbonate solution, and then filtered and air dried to give 4,4′-dimaleimidylstilbene (174 mg; 76% yield). ^1^H NMR (DMSO-d6, 400 MHz) 7.74 (4H, d), 7.38 (4H, d), 7.37 (2H, s), and 7.21 (2, 4H).

### Virus binding and entry assays.

To study virus binding and entry by detection of viral DNA, HFFs were infected at 4°C for 1 h to allow virus binding. The cells were washed in ice-cold phosphate-buffered saline (PBS) and then either lysed immediately for analysis by qPCR for genomes (using UL138 primers as noted below) or temperature shifted to 37°C to promote viral entry. Fifteen minutes postentry, DNA was harvested from the cells and analyzed by qPCR. To assess virus binding by direct visualization of virus particles, HFFs were first labeled with 1 μg/ml Hoechst 33342 (Tocris) for 10 min at 37°C. The cells were then washed in ice-cold PBS and infected at 4°C at a multiplicity of infection (MOI) of 5 with TB40/E-UL32-GFP HCMV for 1 h. The cells were washed twice with ice-cold PBS, and live cells were visualized directly by confocal imaging using a 60× objective on a Nikon Ti inverted microscope with a C2 confocal scan head (Nikon). Z-stacks were acquired at high zoom to create representative images, while z-stacks of multiple low-zoom fields were acquired to allow quantification. ImageJ was used to process images for display.

### Fixation and immunostaining.

Human cells were fixed by treatment with 100% ice-cold ethanol for >20 min at –20°C. Murine 3T3 cells were fixed by treatment with 100% ice-cold methanol for 30 min at –20°C. To detect viral proteins, the cells were then washed in PBS and incubated with mouse anti-IE (6F8.2; Merck Millipore; 1:2,000 dilution), mouse anti-pp28 (5C3; Santa Cruz Biotechnology; 1:1,000 dilution), mouse anti-ICP4 (abcam; 1:1,000 dilution), mouse anti-MCMV IE (Genetex; 1:1,000 dilution) or mouse anti-pp65 (abcam; 1:500 dilution) for 1 h, followed by incubation with goat anti-mouse IgG–Alexa-fluor 568 (Life Technologies; 1:2,000 dilution) plus 0.5 μg/ml DAPI (4′,6-diamidino-2-phenylindole) for 1 h.

### Innate and humoral immunity studies and quantification of infection.

To measure the impact of humoral immunity on HCMV infection, all viruses were preincubated with pooled sera from seropositive or seronegative donors for 1 h at dilutions from 1:5 to 1:800. After 1 h, the cells were infected with these HCMV preparations and fixed and stained 24 hpi for IE gene expression as a marker of lytic infection.

For studies with IFN, cells were pretreated with a combination of IFN-α/β (Peprotech) for 3 h and then infected with HCMV at an MOI of 1. After 16 h, the cells were fixed and stained for IE gene expression as a marker of lytic infection.

Cells stained for viral IE protein expression were quantified by automated fluorescence microscopy and image recognition. A Hermes WiScan (IDEA Bio-Medical) automated microscope was used to image the central 25% of each well (20 images/well), and the resultant images were processed with MetaMorph microscopy automation and image analysis software (Molecular Devices).

### Oligonucleotide design for shRNA production.

Sequences for shRNA production were designed using the RNAi Consortium of the Broad Institute (https://portals.broadinstitute.org/gpp/public/). The selected small interfering RNA (siRNA) sequence for CLCN2 knockdown was CCTTTGCTGTCATTGGTATTG. To engineer the shRNA vectors, the following oligonucleotides were used: CLCN2 (F, GAT CCG CCT TTG CTG TCA TTG GTA TTG TTC AAG AGA CAA TAC CAA TGA CAG CAA AGG TTT TTT G; R, AAT TCA AAA AAC CTT TGC TGT CAT TGG TAT TGT CTC TTG AAC AAT ACC AAT GAC AGC AAA GGC G) and Scramble (F, GAT CCG TTC TAA CAT GAC TCT AGT AAT TCA AGA GAT TAC TAG AGT CAT GTT AGA ACT TTT TTG; R, AAT TCA AAA AAG TTC TAA CAT GAC TCT AGT AAT CTC TTG AAT TAC TAG AGT CAT GTT AGA ACG).

### Cloning of an shRNA-bearing vector.

The pSIREN-RetroQ vector (a gift from Paul Lehner, Cambridge University) was digested with BamHI-HF and EcoRI-HF in CutSmart buffer and dephosphorylated with Antarctic phosphatase (New England Biolabs) according to the manufacturer’s instructions. The product was subjected to gel electrophoresis, and the linear plasmid was purified using a QIAquick gel extraction kit (Qiagen) according to the manufacturer’s instructions.

Oligonucleotides for cloning into the pSIREN-RetroQ vector were annealed and phosphorylated using T4 polynucleotide kinase (T4 PNK) (New England Biolabs). The annealed oligonucleotides were diluted 1:20 with double-distilled water (ddH_2_O). Ligation of the annealed primers into the linearized plasmid was performed using T4 DNA ligase (New England Biolabs) according to the manufacturer’s instructions. Two microliters of the ligation reaction mixture was used to transform 25 μl of Alpha-Select silver efficiency competent Escherichia coli bacteria (Bioline) according to the manufacturer’s instructions and plated onto LB agar supplemented with 100 μg/ml ampicillin. Single colonies were inoculated into LB medium supplemented with 100 μg/ml ampicillin. Insertion of the desired sequence was confirmed by Sanger sequencing (Eurofins).

### Production of shRNA-bearing lentiviruses.

Three microliters of TransIT-293T (Mirus) was mixed with 0.75 μg shRNA-bearing pSIREN-RetroQ vector, 0.5 μg pCMV-dR8.91, and 0.25 μg pCMV-VSV-G in 100 μl Opti-MEM for 20 min at room temperature. The mixture was then added dropwise to 70% confluent 293T cells in a 12-well plate with 500 μl DMEM plus 10% FBS; 8 h later, 500 μl DMEM plus 10% FBS was added. Two days posttransfection, the supernatant was clarified by centrifugation and stored.

### Transduction of HFFs with lentiviral vectors and selection of transduced cells.

Five hundred microliters of lentivirus was added to 60% confluent HFFs in a 12-well plate in a 1.5-ml total volume. The plates were then spun at 200 × *g* for 30 min. Two days postransduction, transduced HFFs were selected with 1 μg/ml puromycin for 2 days. Successful knockdown of CLCN2 was confirmed by quantitative reverse transcription (qRT)-PCR.

### Nucleic acid isolation and analysis.

DNA was extracted by proteinase K digestion. Cells were treated with 100 mM KCl, 10 mM Tris-HCl, and 2.5 mM MgCl for 5 min prior to the addition of an equal volume of 10 mM Tris-HCl, 2.5 mM MgCl, 1% (vol/vol) Tween 20, 1% (vol/vol) Nonidet P-40 (Santa Cruz Biotechnology), and 0.4 mg/ml proteinase K (Sigma-Aldrich) for a further 5 min. The resultant mixture was heated for 1 h at 60°C, followed by 10 min at 95°C.

RNA was extracted using a Qiagen RNeasy kit, with columns from Epoch Life Sciences, according to the manufacturer’s instructions. cDNA was then synthesized from 250 ng total RNA using a Qiagen Quantitect reverse transcription kit according to the manufacturer’s instructions.

Relative quantification by qPCR was performed using PowerUp SYBR green master mix (ThermoFisher) with forward and reverse primers (250 nM) according to the manufacturer’s instructions, using an Applied Biosystems 7500 real-time PCR system (50°C for 2 min; 95°C for 2 min; 40 cycles of 95°C for 15 s, 60°C for 15 s, and 72°C for 1 min). The data were analyzed by the ΔΔ*C_T_* method using 18S RNA as a housekeeping gene. The following gene-specific primers (Invitrogen) were used: 18S (F, GTA ACC CGT TGA ACC CCA; R, CCA TCC AAT CGG TAG TAG CG), UL138 (F, GAG CTG TAC GGG GAG TAC GA; R, AGC TGC ACT GGG AAG ACA CT), IFIT2 (F, ACT GCT GAA AGG GAG CTG AA; R, TGC ACA TTG TGG CTT TGA AT), IFIT3 (F, AGA AAT GAA AGG GCG AAG GT; R, ATG GCC TGC TTC AAA ACA TC), CXCL10 (F, TGG CAT TCA AGG AGT ACC TC; R, TTG TAG CAA TGA TCT CAA CAC G), and CLCN2 (F, GAT CGT GTT CGA GCT CAC AG; R, GCA GGT AGG GCA GTT TCT TG). Absolute quantification by qPCR for viral genome replication was performed using TaqMan Fast Advanced master mix (ThermoFisher) with cycling conditions of 50°C for 2 min and 95°C for 10 min, followed by 40 cycles of 95°C for 3 s and 60°C for 30 s. Primers against gB (F, GAG GAC AAC GAA ATC CTG TTG GGC A; R, TCG ACG GTG GAG ATA CTG CTG AGG) were used at 100 nM final concentration. The product was detected by TaqMan probe (CAA TCA TGC GTT TGA AGA GGT AGT CCA CG) labeled with 6-carboxyfluorescein (FAM) and 6-carboxytetramethylrhodamine (TAMRA) fluorophores. Samples containing known quantities of viral genomes were included to allow absolute quantification.

### Construction of HCMV mutants.

HCMV mutants were made by BAC recombineering based on the method of Stanton et al. (50). BACgMmut was generated from BACgMwt (RCMV1778), while BAC_RL13trunc was generated from BAC_RL13wt (RCMV1516). BAC_RL13trunc+gMmut was then generated from BAC_RL13trunc. Oligonucleotide sequences for amplification of an *rpsL* selection cassette for insertion of the gM mutation were as follows: F, GCT CAC TCT TTT TCT TCT CGC GTC TGC ACC CCA AGC TCA AAG GTA CGG TGC AGT TCC GCA CGC TCA TCG TCA ACC TGG CCT GTG ACG GAA GAT CAC TTC G, and R, CGC ACG AAA AAG TTG TTT CCG AAG CCG TAG CAC AGG GCC ATG GCT ACC ACG GTG GTG TTG AAA CCA AGC GCT ACC TCT ACT GAG GTT CTT ATG GCT CTT G. Oligonucleotide sequences for amplification of the cassette for insertion of RL13 truncation were as follows: F, ACG CGA TTT GAA TAT AAT ATC ACG GGA TAT GTT GGC CAA GAA GTG ACT CTA AAC TTC ACT GGA TCA TGG AAT TAC ATT CCT GTG ACG GAA GAT CAC TTC G, and R, GTA TGA TGA TAA TTG CTG GTA ACG GTG CAT ATT GGT TCC GAG GAA TAA AGC CAG CCT GGA GAA CCG TAC CGG AAC CAT TCT GAG GTT CTT ATG GCT CTT G. The oligonucleotide sequence for insertion of gM mutation was CCC CAA GCT CAA AGG TAC GGT GCA GTT CCG CAC GCT CAT CGT CAA CCT GCT AGA GGT AGC GCT TGG TTT CAA CAC CAC CGT GGT AGC CAT GGC CCT GTG C and for RL13 was ATG TTG GCC AAG AAG TGA CTC TAA ACT TCA CTG GAT CAT GGA ATT ACA TTT AAT GGT TCC GGT ACG GTT CTC CAG GCT GGC TTT ATT CCT CGG AAC CAA T.

### Library preparation, deep sequencing, and assembly.

Illumina sequence libraries were constructed by combining a NEBNext Ultra II DNA library preparation kit (NEB; E7645) and Agilent SureSelect XT kits. Here, 200 ng of total DNA, derived from original DNA extracts and supplemented with human DNA (Promega; G1471), was used as input for each sample. The DNA was sheared using a Covaris E220 (peak incident power, 175; duty factor, 5; cycles per burst, 200; treatment time, 150 s), and the fragmented DNA was used as input for NEBNext end preparation, as described in the manufacturer’s instructions. Adaptor ligation was performed as described in the instructions, except that a 1:10 dilution of the SureSelect adapter was used and the USER enzyme step was omitted. Twelve cycles of PCR-based amplification of the end-repaired and adapter-ligated library were carried out as described in the SureSelect XT protocol (version C0; December 2016). Hybridization-based enrichment of HCMV sequences was undertaken using a custom HCMV bait set (based on the HCMV reference strain Merlin) for 24 h, as described in the instructions. Sequence libraries were multiplexed and run on an Illumina MiSeq (two 300-bp paired-end reads).

Following the sequencing run, paired-end sequence data were demultiplexed, and sequence reads were trimmed to remove low-quality 3′-based and adapter sequences using TrimGalore (http://www.bioinformatics.babraham.ac.uk/projects/trim_galore/). Read pairs were subsequently aligned against the HCMV reference strain Merlin (NC_006273.2) using BBMap (http://sourceforge.net/projects/bbmap/), while duplicate read pairs were removed using MarkDuplicates (http://broadinstitute.github.io/picard), and local realignment was performed using IndelRealigner (53). SAM, BAM, and mpileup files were parsed using SAMTools v1.0 (54), VarScan 2 (55), and custom PERL scripts to produce both a consensus sequence for each sample and frequency profiles for sites with two or more alleles present.

### Generation of DIDS-resistant virus.

To generate a DIDS-resistant strain of HCMV, HFFs were infected with the clinical Merlin strain at a low MOI in the presence of 10 μM DIDS. The virus was allowed to propagate through the cell layer, with the medium and DIDS refreshed every 3 or 4 days, until all the cells exhibited cytopathic effect. Supernatants or physically disrupted cell layers were transferred onto fresh HFFs in the continued presence of DIDS. The process was repeated over a period of 6 months, with the concentration of DIDS gradually escalating to a maximum of 100 μM. The concentration of DIDS was then reduced to 25 μM to allow production of sufficient levels of virus for assessment.
